# Integrative profiling of untreated primary membranous nephropathy at the single-cell transcriptome level

**DOI:** 10.1093/ckj/sfae168

**Published:** 2024-06-14

**Authors:** Qiuhua Gu, Yuchen Wen, Xi Cheng, Yan Qi, Xin Cao, Xiqian Gao, Xiaoming Mao, Wenya Shang, Li Wei, Junya Jia, Tiekun Yan, Zhigang Cai

**Affiliations:** Department of Nephrology, Tianjin Medical University General Hospital, Tianjin, China; National Key Laboratory of Experimental Hematology, Tianjin, China; The Province and Ministry Co-Sponsored Collaborative Innovation Center for Medical Epigenetics, Department of Pharmacology, School of Basic Medical Science, Tianjin Medical University, Tianjin, China; Department of Nephrology, Tianjin Medical University General Hospital, Tianjin, China; Department of Nephrology, Tianjin Medical University General Hospital, Tianjin, China; Department of Nephrology, Tianjin Medical University General Hospital-Airport Hospital, Tianjin, China; Department of Nephrology, Tianjin Medical University General Hospital-Airport Hospital, Tianjin, China; Department of Nephrology, Tianjin Medical University General Hospital, Tianjin, China; Department of Nephrology, Tianjin Medical University General Hospital, Tianjin, China; Department of Nephrology, Tianjin Medical University General Hospital, Tianjin, China; Department of Nephrology, Tianjin Medical University General Hospital, Tianjin, China; Department of Nephrology, Tianjin Medical University General Hospital, Tianjin, China; National Key Laboratory of Experimental Hematology, Tianjin, China; The Province and Ministry Co-Sponsored Collaborative Innovation Center for Medical Epigenetics, Department of Pharmacology, School of Basic Medical Science, Tianjin Medical University, Tianjin, China; Tianjin Key Laboratory of Inflammatory Biology, Tianjin, China

**Keywords:** B cells, podocytes, primary membranous nephropathy, single-cell RNA sequencing, T cells

## Abstract

**Background:**

Primary membranous nephropathy (PMN) is an autoimmune kidney disease. Despite the identification of certain autoantigens, the etiology and pathophysiology of PMN are still largely unknown.

**Methods:**

Five patients with biopsy-proven PMN were enrolled in this study. Their blood, kidney and urine samples were collected respectively to profile cellular, molecular and immunological alterations by using single-cell RNA sequencing (scRNA-seq). Experimental verifications were also implemented in kidney tissue.

**Results:**

In the peripheral blood mononuclear cell (PBMC) samples, portions of B cells and plasma cells were increased in PMN patients. Cell–cell communication analysis suggests that *APRIL* (a proliferation-inducing ligand from B cells) might be a potential molecule that regulates the activity of plasma cells. In the kidney samples, scRNA-seq analysis showed that the infiltration of T cells, as well as the myeloid cells, appears abundant compared with healthy controls, suggesting that immune cells are actively recruited to kidney. Furthermore, we observed an enhanced interaction between inflammatory cells and podocytes, which might contribute to kidney injury. Accordingly, scRNA-seq analysis of urinary samples is partially reminiscent of the kidney cell landscape, especially T cells and myeloid cells, suggesting monitoring urinary samples is a promising method to monitor PMN development. Additionally, integrative analysis across the blood, kidney and urine identified *LTB, HERP1, ANXA1, IL1RN* and *ICAM1* as common regulators of PMN. Finally, immune repertoire in PBMC also showed an elevated diversity of clonal type, implying the existence of autoreactive T-cell receptor/B-cell receptor.

**Conclusion:**

Our study comprehensively profiled the transcriptomic landscapes of blood, kidney and urine in patients with PMN using scRNA-seq. We depicted the alterations including cell compositions and cell–cell communication in PMN. These results offer important clues with regard to the diagnosis and pathogenesis of PMN and potential intervention of PMN progression.

KEY LEARNING POINTS **What was known:**

- Primary membranous nephropathy (PMN) is an autoimmune kidney disease, with circulating autoantibodies targeting the podocytes, resulting in the accumulation of immune complexes on the sub-epithelial layer of the glomerular capillary wall and kidney injury.

**This study adds:**

- Using single-cell RNA sequencing, we found: portions of B cells and plasma cells were significantly increased in the peripheral blood mononuclear cells, with *APRIL* as a potential regulator.
- Kidney infiltration of T cells and the myeloid cells appeared abundant, similar to that of urinary samples, and obvious upregulation of signaling molecules on podocytes was notable.
- Several genes as common regulators of PMN were identified by integrative analysis across the blood, kidney and urine.

**Potential impact:**

- Offering important clues for the diagnosis and pathogenesis of PMN.
- Providing potential intervention targets for the PMN progression.

## INTRODUCTION

Membranous nephropathy (MN) is the leading cause of nephrotic syndrome in adults, characterized by the accumulation of immune complexes on the sub-epithelial layer of the glomerular capillary wall, which results in the thickened glomerular basement membrane. The immune deposits are formed by the autoantibodies binding to the antigens on the podocyte, which results in complement activation and glomerular capillary injury. Recent studies have identified phospholipase A2 receptor (PLA2R) as the main target antigen in podocytes of patients with primary MN (PMN) [1]. Circulating anti-PLA2R antibody is detected in about 70% of patients with PMN [1]. Recently, considerable advances in the identification of new podocyte antigens [2] and immunological pathogenesis of PMN have been achieved [3]. Furthermore, rituximab has been demonstrated as a new efficacious treatment [4]. However, in the real-world clinic setting there are still some patients who are resistant to various treatment regimens, and go on to develop renal failure [5]. Thus, a comprehensive understanding of the immune mechanism that drives PMN is needed to identify new treatment targets. In addition to some known risk genetic factors for PMN [6–8], both autoantibodies [9, 10] and T cells [11] are suggested to be involved in the pathogenesis of PMN, resulting in the complement activation [12–14] and subsequently podocyte injury. The abnormal activation of immune response suggests that various factors could contribute to the development of PMN and need to be explored, especially the role of each type of immune cells and stromal cells, and also the interactions among the cells and the key regulators of PMN.

Single-cell RNA sequencing (scRNA-seq) technologies are becoming a promising approach to detecting gene expression of individual cells in complex tissue (diseased or healthy), providing a powerful and landscape-scale tool to analyze the cellular compositions, cell–cell communications, molecular pathways, and mechanisms of cellular and molecular alterations underlying pathogenesis of diseases, including kidney diseases [15].

Application of scRNA-seq to explore the kidney changes of the patients with PMN has been reported using minimal samples [16]. More recently, by mapping the cells in the urine of patients with PMN using scRNA-seq, Liu *et al*. found the function and origin of urinary macrophages were different in patients with complete remission and no response to the treatment [17]. However, a more comprehensive analysis of cells in the blood, kidney and urine of patients with PMN using paired samples has not been reported yet. In the current study, we collected blood, kidney tissue and urine samples (five PMN patients and four healthy donors), and also included published scRNA-seq datasets from six PMN patients (GSE171458), and then performed scRNA-seq technologies on the three different types of clinical samples to depict alterations with regard to the cell composition, cell–cell cross-reactions and the potential pathways in the blood, kidney and urine of patients with PMN. Our results profile the landscape alterations in PMN and offer new resources and therapeutic targets for future studies.

## MATERIALS AND METHODS

### Patients

The present study was in compliance with the Declaration of Helsinki and approved by the Ethics Committee of Tianjin Medical University General Hospital. Informed consent from each patient was obtained for samples of blood, urine and kidney. Five patients with anti-PLA2R antibody positivity and biopsy-proven PMN were enrolled at the Department of Nephropathy of Tianjin Medical University General Hospital from December 2021 to April 2023. Pathological diagnosis was obtained according to the manifestations on immunofluorescence, light and electron microscopy (Supplementary data, Fig. S1), according to the standard procedure in our hospital [18]. Clinical and pathological parameters were routinely documented at enrollment. All the patients had circulating anti-PLA2R antibody (Table 1) and were also positive for PLA2R in kidney tissue (Supplementary data, Fig. S1D), which were detected according to the description in our previous study [18]. Secondary factors that potentially induce MN were excluded according to patients’ history, thus the patients in present study could be recognized as PLA2R-associated PMN.

**Table 1:** Clinical and pathological parameters of patients with PMN.

	P1	P2	P3	P4	P5
Samples					
Kidney tissue	✓	✓	✓		
Peripheral blood	✓	✓	✓		
Urine			✓	✓	✓
Clinical parameters					
Gender (F/M)	F	M	M	M	F
Age (years)	33	66	57	33	34
Urinary protein (g/24 h)	7.5	12.0	6.9	4.0	5.5
Serum albumin (g/L)	15	21	19	22	20
Serum creatinine (µmol/L)	44	141	66	65	42
Anti-PLA2R antibody (RU/mL)	65.7	1784.0	216.9	266.0	62.7
Pathological parameters					
MN stage	Ⅱ–Ⅲ	Ⅱ	Ⅱ–Ⅲ	Ⅱ–Ⅲ	Ⅲ
IgG deposition	4+	4+	4+	4+	4+
IgA deposition	–	–	2+	–	–
IgM deposition	2+	1+	1+	2+	2+
C3 deposition	2+	2+	2+	2+	2+
C1q deposition	–	–	+	–	–
PLA2R staining	+	+	+	+	+
Crescent formation	0/8	0/13	0/9	0/14	0/19
Glomerular sclerosis	0/8	2/13	0/9	0/14	0/19
Chronic tubulointerstitial injury (%)	<25	25–50	<25	<25	<25
Interstitial infiltration (%)	<25	25–50	<25	<25	<25

P1–5, patients 1–5; F, female; M, male.

### Sample preparation

**Figure 1:**
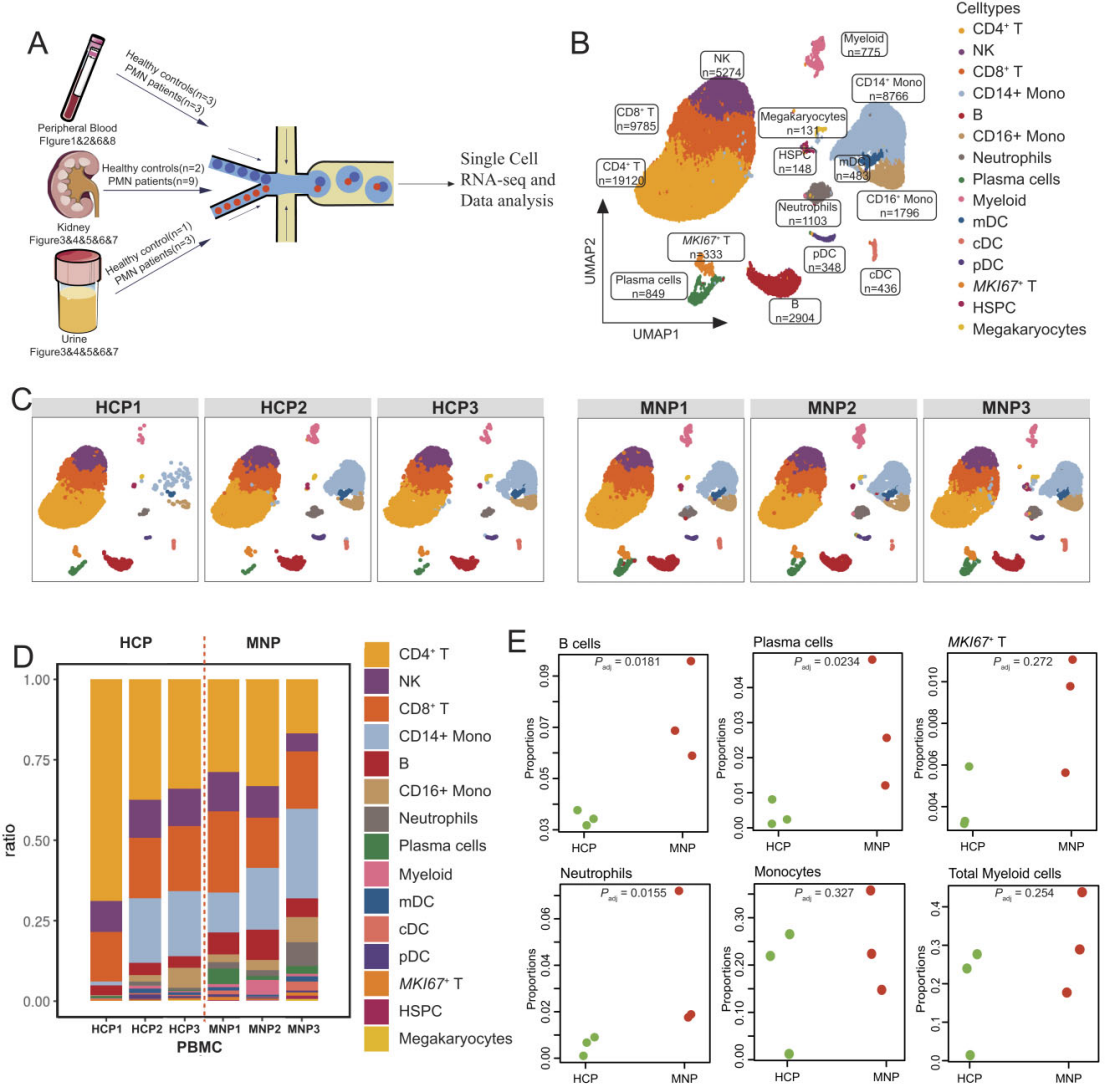
scRNA-seq analysis of PBMCs from patients with PMN and healthy controls. (**A**) Overview of the experimental workflow and numbers of patients used in the analysis. (**B**) UMAP plot for 52 251 high-quality single cells from PBMC samples, annotated with 15 cell types. (**C**) Individual UMAP visualization of PBMC from three healthy controls (HCP1, HCP2, HCP3) and three patients (MNP1, MNP2, MNP3). (**D**) The stacked bar chart showing the proportions of different cell types in each sample. (**E**) The dotplot showing the proportions of indicated lymphoid and myeloid cells in patients and controls.

The study was performed as shown in Fig. 1A. Kidney biopsy tissues were obtained from three patients with PMN. After harvested, fresh kidney samples were washed in ice-cold RPMI1640 and then dissociated using Collagenase II (Sigma, V900892-100MG) and DNase I (Sigma, DN25-1 G). Peripheral blood from three patients with PMN and three healthy controls were also collected. Fresh blood was separated to obtain peripheral blood mononuclear cells (PBMCs) using Histopaque-10771 (Sigma-Aldrich, catalog No.10771-6 ×100 mL) following the procedure of instruction. Urine samples were obtained from three PMN patients and one healthy control. Urine (300–900 mL) after the first morning void was collected and then was filtered using a 70-μm cell filter (Miltenyi 130-098-462), and centrifuged at 300*g*, 4°C for 5 min to collect cell precipitates.

Cell count and viability were estimated using fluorescence Cell Analyzer (Countstar^®^ Rigel S2) with AO/PI reagent after removal erythrocytes (Solarbio R1010). Finally, fresh cells were washed twice in the RPMI1640 and then resuspended at 1 × 10^6^ cells/mL in 1× phosphate-buffered saline (PBS) containing 0.04% bovine serum albumin.

### Single-cell sequencing

Single-cell RNA-Seq libraries were generated using the SeekOne^®^ MM Single Cell 3′ library preparation kit following established protocols as described in the procedure of instruction. In brief, an appropriate quantity of cells was introduced into the flow channel of the SeekOne^®^ MM chip, which was equipped with 170 000 individual microwells, facilitating the cellular settlement through gravitational forces. After eliminating non-settled cells, sufficient Cell Barcoded Magnetic Beads (CBBs) were meticulously added to the flow channel and subsequently allowed to settle within the microwells under the influence of a magnetic field. Following this, surplus CBBs were thoroughly washed out, and the cells confined within the SeekOne^®^ MM chip were subjected to lysis, liberating RNA molecules. The released RNA was then effectively captured by the CBB present within the corresponding microwell. Following this, all the CBBs were collected and a reverse transcription process was carried out at 37°C for a duration of 30 min, enabling the labeling of cDNA with a cell-specific barcode attached to the beads. After the reverse transcription, an Exonuclease I treatment was executed, effectively eliminating any unutilized primers remaining on the CBBs. Consecutively, the barcoded cDNA immobilized on the CBBs was subjected to a hybridization process with a random primer featuring a SeqPrimer sequence on its 5′-terminus, thus enabling its extension to form the second strand DNA. This extension led to the integration of the cell barcode onto the 3′-terminus of the DNA strand. The resultant second strand DNA was then denatured and removed from the CBBs, after which a purification step was undertaken. The purified DNA was subsequently subjected to amplification through a polymerase chain reaction (PCR) reaction. The amplified cDNA product was then subjected to a purification process to eliminate extraneous fragments, following which it was incorporated with a full-length sequencing adapter and a sample index through an indexed PCR procedure. The resulting indexed sequencing libraries underwent a cleanup process using SPRI beads, were quantified using quantitative PCR (KK4824, KAPA Biosystems), and were then subjected to sequencing on an Illumina NovaSeq 6000 with PE150 read length.

### Data processing of scRNA-seq libraries

Demultiplexing, barcode processing, read alignment, and gene expression quantification and filtration were carried out using SeekSoul-tools software (v1.2.0, SeekGene). Both matrix files and fastq files were provided for downstream analysis.

### scRNA-seq data analysis

Data analysis were mainly performed in R (4.2.0) and LINUX OS. The coding environment has been described in our previous study [19]. Briefly, Seurat (version 4.3.0) was used for downstream analysis. Data from each patient were imported using the Read10× function and then structured into a Seurat object using CreateSeuratObject. For quality control, cells with unique feature counts >2000 or <200, and cells with >20% mitochondrial proportions were calculated and filtered out. Data were normalized using LogNormalize and scaled to prepare for linear dimensional reduction. After principal component analysis (“RunPCA” function, dims 1:20), integration and batch correction were done by Harmony (“RunHarmony” function). Dimensional reduction by uniform manifold approximation and projection (UMAP) (dims = 1:20) and clustering was performed using reduction = harmony. Cluster was determined with resolution = 0.6 and cell type annotation was adjusted accordingly. For example, cell types were defined based on differential gene expression as determined by the Seurat FindAllMarkers function. Cell compositions were quantified by the frequency of certain cell types in the context.

### Cell proportion analysis

*Propeller* [20] is a function in the Speckle v.0.0.3 that uses cell-level annotation information to calculate sample-level cell-type proportions, followed by data transformation and statistical testing for each cell type in PBMC. The parameter transform=‘logit’ in *propeller* is used to calculate significance.

### Cellular pathway score

ClusterProfiler v.4.6.2 was used for enrichment analysis in R. Add ModuleScore_UCell was used for pathway scoring. All cell types were included in the score. ggplot2 were used to visualize the results and the hallmark gene set [21] in the Human Molecular Signatures Database (MSigDB) was selected as the pathway. The stat_compare_means function was used to calculate the difference between the two groups, using the t.test method.

scGSVA v0.0.16 [22] was used to perform enrichment scores for Kyoto Encyclopedia of Genes and Genomes (KEGG) pathways. We used the buildAnnot function to retrieve the KEGG pathway dataset and applied the scgsva function to perform the UCell score. The sigPathway function is used to screen for significant pathways between groups. vlnPlot display the filtering pathway, and the above method is also used to calculate the significance.

### Cell communication analysis

Cell–cell interaction analysis was performed using CellChat v.1.4.1 [23] based on the curated ligand-receptor interaction database (CellChatDB) in R. All cell types in PBMCs were included in the analysis. In kidney tissue and urine, only immune cells and podocytes were included for analysis and comparison. First, the subset function is used to group healthy individuals and patients to construct a CellChat input file on a normalized gene expression matrix. The total numbers of interactions and interaction strengths were computed by the computeCommunProb function, and the communication probabilities for each cell signaling pathway were calculated by computeCommunProbPathway function. Then, mergeCellChat function was used to merge the two sets of data for comparison.

NicheNet v.2.0.0 [24] was used to identify potential ligands that drive the phenotype of PMN. The top 20 differentially expressed genes (DEGs) were ordered using log2FC between neutrophils, and the remaining clusters were treated as potential targets. Genes with a positive rate of >10% were considered background and potential ligands, respectively. Sender cells from all cell types in PBMC (only immune cells and podocytes in kidney and urine) and target cells from neutrophils were included to construct the expressed ligand–receptor interactions and calculate the ligand activity. We used the active_ligand_target_links function to compute the potential intensity of regulation between the ligand and target.

### DEGs analyses

We calculated DEGs for each cell type separately between patients and healthy individuals. In the DEG analyses, the FindMarkers function was used from the Seurat package.

### Gene Ontology pathway analysis

The ClusterProfile.v.4.6.2 R package's EnrichGO function was used to analyze Gene Ontology (GO) terms. Molecule function, biological process and cellular component are the three parts of GO analyses. We included all these three parts in the study. Visualization of the results was done using ggplot2.v 3.4.2 in R.

### Gene expression correlation

For gene expression correlation (normalized mean expressions for the top 500 variable genes) between kidney and urine were calculated per cluster. Correlations were calculated using the package rcorr and visualized using the package corrplot. Gene co-expression networks were further analyzed in kidney and urine. We inferred an important gene co-expression network with the R package BioNERO.v.1.9.5 [25] and compared the differences between the two tissues.

### Analysis of the immune repertoire

Based on single-cell RNAseq bam files, the TRUST4 [26] software version 1.0.12 was used to determine the landscape of immune cells in PBMC. T-cell receptor (TCR) and B-cell receptor (BCR) clonality was analyzed using the R package immunarch.v.0.9.0 [27].

### Detection of T cells and macrophages in the kidney

The paraffin-embedded kidney tissue of the five patients with PMN and three patients with minimal change disease (MCD) served as control were used to detect the infiltration of T cells and macrophages. Briefly, the renal biopsy specimens were deparaffinized, hydrated and subjected to heat treatment for antigen retrieval. Then all the sections were incubated respectively with rabbit anti-human CD3 and CD68 antibodies (ZSJQ-Bio, Beijing, China) at 4°C overnight, followed by incubation with anti-rabbit immunoglobulin conjugated with horseradish peroxidase (ZSJQ-Bio, Beijing, China) at 37°C for 30 min, and the staining was visualized with 3,3′-diaminobenzidine. Primary antibodies were replaced by PBS as negative controls. Sections were examined by light microscopy, and not fewer than five random areas of renal tissues under a 200-fold magnification were analyzed. The infiltration of T cells was described as CD3^+^ cells every low power field (LP). The staining of CD68 was analyzed with ImageJ and expressed as integrated optical density every LP.

### Statistical analysis

Clinical samples in this study were collected from individuals independently. Representative data were shown in the experimental verification sets. Statistical analysis was conducted by using GraphPad 9 or in R using proper packages. Differences of quantitative parameters were assessed using the *t*-test for data that were normally distributed, or nonparametric test for data that were not normally distributed. If not stated elsewhere, results with *P* < .05 were considered statistically significant.

## RESULTS

### Demographic, clinical and pathological parameters of patients with PMN

Five patients with PMN (two females and three males) were enrolled in the study. The average age of them was 44.6 ± 15.8 years. And all of them presented with nephrotic syndrome, with a high level of urinary protein (7.2 ± 3.0 g/24 h) and a low level of serum albumin (19.4 ± 2.7 g/L). Four of them had a normal level of serum creatinine, while one patient showed acute kidney injury with a mildly high level of serum creatinine. Circulating anti-PLA2R antibody (median 216.9, range 64.2–1025.0 RU/mL) could be detected among all the patients. Details of the clinical features are shown in Table 1.

On light microscopy, the classical presence of MN was detected in all the kidney specimens, with MN stage II and III (Supplementary data, Fig. S1B and C). No crescents formation was found in the glomeruli, and the glomerular sclerosis was rare. The chronic tubulointerstitial injury and interstitial infiltration were mild in four patients while the last one patient was severe. Through immunofluorescence microscopy, all five patients showed a strong immunoglobulin G (IgG) staining, with IgG4 as the dominant subclass (Supplementary data, Fig. S1A, E–H), accompanied by deposition of complement 3 and IgM, with or without that of IgA. Notably, the PLA2R staining was positive on all the kidney specimens from the five PMN patients (Supplementary data, Fig. S1D). Table 1 summarizes pathological parameters in detail.

### Cell composition and communication in PBMCs from patients with PMN revealed by scRNA-seq

Single-cell RNA sequencing technologies offer an unprecedent and insightful perspective into the individual cells within the blood, kidney and urine tissues of both PMN patients and healthy controls (Fig. 1A). PBMCs were filtered and subjected for scRNA-seq analysis (see Materials and methods). Following rigorous quality control protocols, we meticulously curated a total of 52 251 high-quality cells, comprising 26 870 cells from patients with PMN and 25 381 cells from healthy individuals. We presented the overall peripheral blood profile as well as the split profile for each individual (Fig. 1B and C). By leveraging classical marker genes elucidated in the literatures, we annotated the cell clusters and identified 15 cell types in PBMCs, including CD4^+^ T cells (*IL7R*), NK cells (*NKG7*), CD8^+^ T cells (*CD8A*), CD14^+^ monocytes (*CD14*), CD16^+^ monocytes (*FCGR3A*), B cells (*MS4A1*), neutrophils (*FCGR3B*), plasma blast cells (*IGKC*), myeloid cells (*LYZ*), mDC (*CD1C*), cDC (*FCER1A*), pDC (*LILRA4*), *MKI67*^+^ T cells (*MKI67*), hematopoietic stem and progenitor cells (HSPC) (*CD34*) and megakaryocytes (*PPBP*) (see Supplementary data, Fig. S2A and B for the expression profiles). In our cohort, we observed that PMN individuals had a significant increase in B cells (*P* = .0181) and plasma cells (*P* = .0234) proportions compared with healthy controls (Fig. 1D and E), which is consistent with previous flow cytometry studies [28, 29]. Additionally, the neutrophils proportion was also signigicantly elevated (*P* = .0155) (Fig. 1D and E). Notably, we also noticed that there were proportional increments in *MKI67^+^* T cells, and myeloid cells (Fig. 1D and E). The cell composition analysis suggests that PMN patients readily manifest increased portion of immune cells in blood, including B cells, plasma cells and neutrophils, which could be partially revealed by both flow cytometry and scRNA-seq analysis.

**Figure 2:**
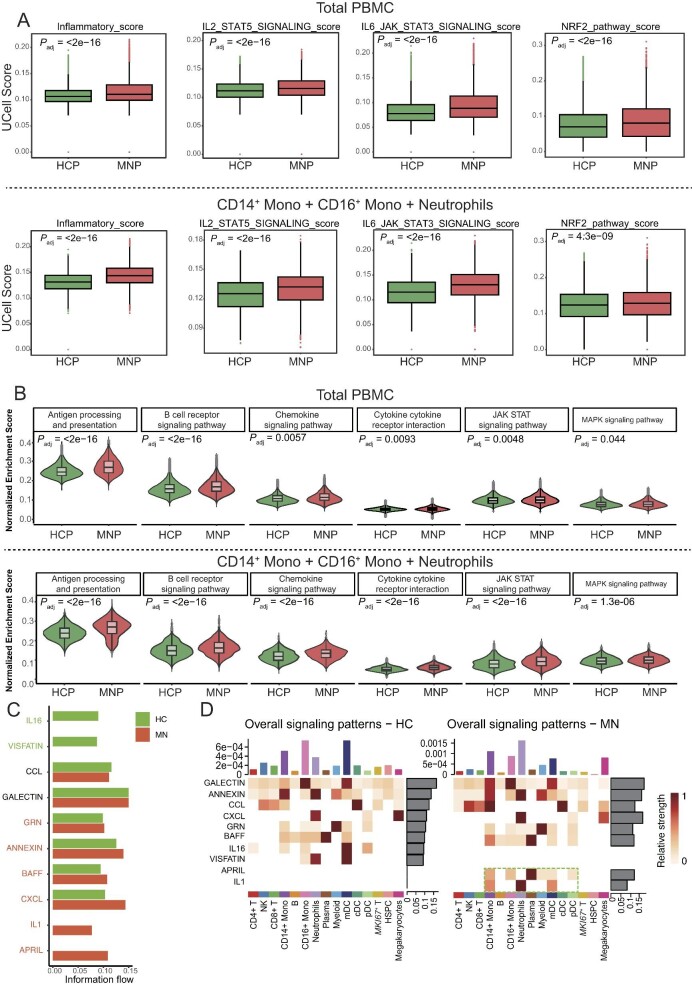
The altered pathways and cell communications in PBMC from patients with PMN. (**A, B**) The box and violin plots visualizing the pathways that were significantly enriched in PMN patients compared with healthy individuals. The involved pathway gene sets were obtained from the GSEA database. (**C**) The bar plot displays the differences in information flow of cell–cell communication between patients and healthy individuals. (**D**) The heatmaps depict the differences in the overall signal patterns between these two groups, as spitted by cell types. Note the dashed lines highlight increased *IL1* signals from neutrophils, monocytes and pDC, and *APRIL* signals from monocytes and mDC in PMN patients. **Adjusted *P *< .05, ***adjusted *P *< .01, ****adjusted *P *< .001.

We compared numerous pathway activities in hallmark gene sets between PMN patients and healthy controls in all cell types and myeloid cells (including CD14^+^ monocytes, CD16^+^ monocytes and neutrophils). The cellular pathway score analysis revealed a significant increase in inflammatory signals in PMN patients, especially in myeloid cells (Fig. 2A). Additionally, utilizing the Gene Set Variation Analysis, we also revealed that antigen-antibody response, B cell–mediated responses and chemokine signaling pathways are increased in PMN patients (Fig. 2B). Quantification of cell–cell communication information flow revealed that obvious alterations were associated with increased ligand *APRIL* (a proliferation-inducing ligand from B cells) and ligand *IL1* while the information flow mediated by ligand *IL16* and *VISFATIN* (a mediator of inflammation) was decreased in patients with PMN, compared with the healthy control (Fig. 2C). Particularly, *APRIL* signaling was activated across six cell types, with a pronounced presence in plasma cells; *IL1* signaling was activated across three myeloid cell types (Fig. 2D), indicating the proinflammatory environment in the circulation of patients with PMN. Additional cell–cell communication analysis suggests that plasma cells might interact strongly with dendritic cells and monocytes through *APRIL* as an input signal (Supplementary data, Fig. S3A–D). However, *VISFATIN* is mainly transmitted from neutrophils to mDC cells in healthy controls. In summary, the cell–cell communication analysis indicates that scRNA-seq technologies systematically detected profound alterations in immune cells, particularly mediated by *APRIL* and *IL1* information flow in patients with PMN compared with healthy controls.

### Single-cell transcriptome profiles of kidney biopsy and urine samples from patients with PMN

**Figure 3:**
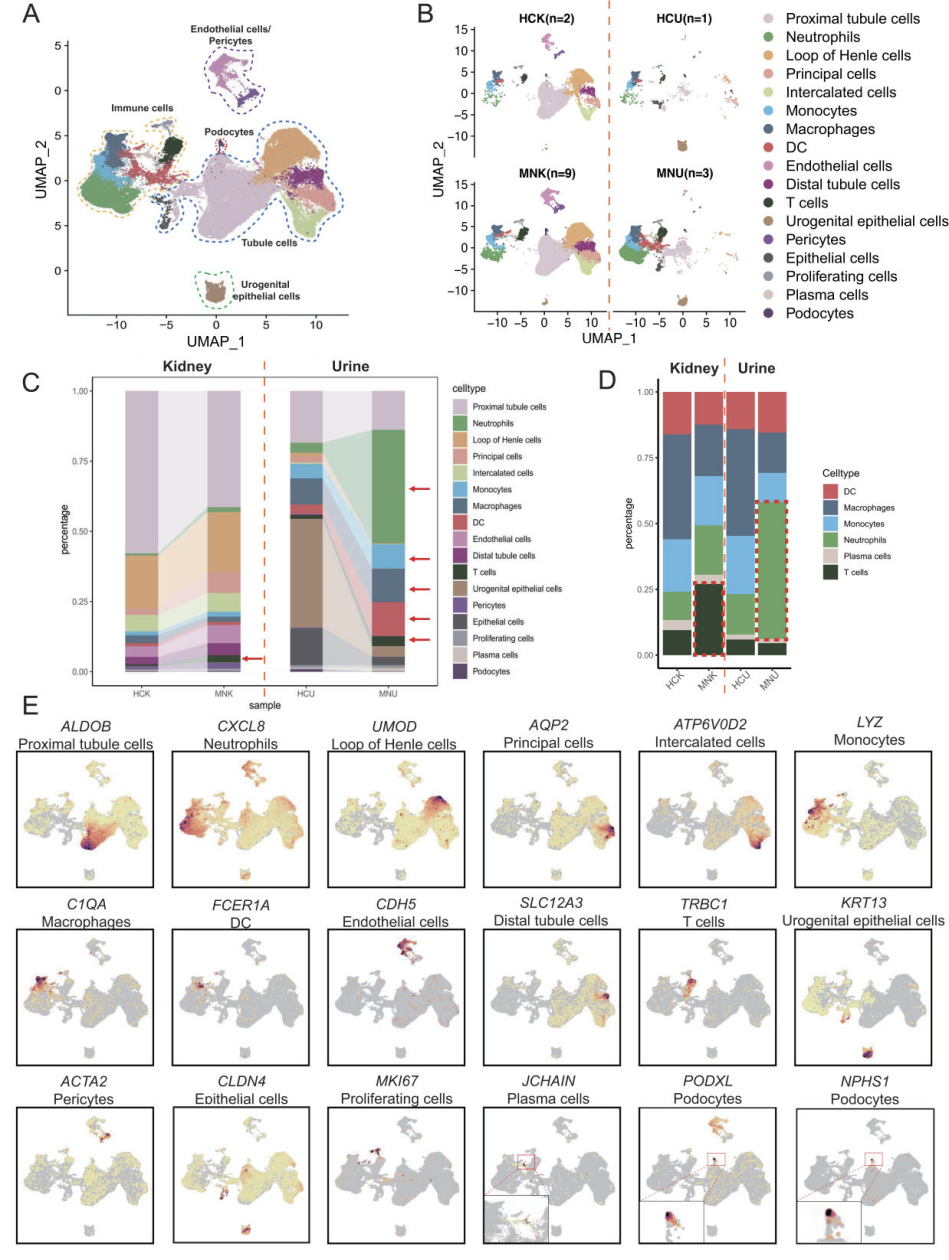
scRNA-seq analysis of kidney and urine from patients with PMN and healthy controls. (**A**) UMAP plot for 17 cell types of 66 552 high-quality single cells from kidney and urine samples. Nine patients and two healthy controls are with kidney samples. Three patients and one healthy control are with urine samples. (**B**) Individual UMAP plots from the four groups (HCK, MNK, HCU and MNU). (**C**) Stacking bar plot showing the proportions of the 17 cell types in four groups. (**D**) Stacking bar plot showing the proportions of only immune cells in four groups. (**E**) Feature plot illustrating expression of canonical markers of the 17 cell types.

Subsequently, we carried on the acquisition of single-cell transcriptome profiles from kidney and urine samples sourced from both patients with PMN and healthy controls. Due to rare donors of healthy kidney biopsy tissue in our center, we integrated publicly available scRNA-seq datasets of kidney tissues from two healthy individuals into three PMN patients of the present study [16]. To improve the statistical power of the present study, we also included the published scRNA-seq datasets of kidney tissues from six PMN patients with similar clinical features to the current study in China (thus the number of PMN patients with kidney scRNA-seq increased to nine) [16]. For the convenience of subsequent studies, we also combined the scRNA-seq datasets of the kidney tissues with that of urine samples. After rigorous quality control procedures, we presented a cell atlas of human kidney and urine (Fig. 3A and 3B). In the atlas, we yielded 47 123 high-quality cells from kidney tissue (two healthy controls have 9416 cells; nine PMN patients have 37 707 cells) (Fig. 3A and 3B). Urine samples from only one healthy individual comprised 2337 cells, while those from three PMN patients consisted of 17 092 cells (Fig. 3A and 3B). Figure 3B displays the UMAP maps of all samples from the four groups. Similar to the data processing in PBMCs, we meticulously annotated the heterogeneous cell population originating from both kidney and urine samples and identified 17 distinct cell types (Fig. 3E, Supplementary data, Fig. S4A) [16].

**Figure 4:**
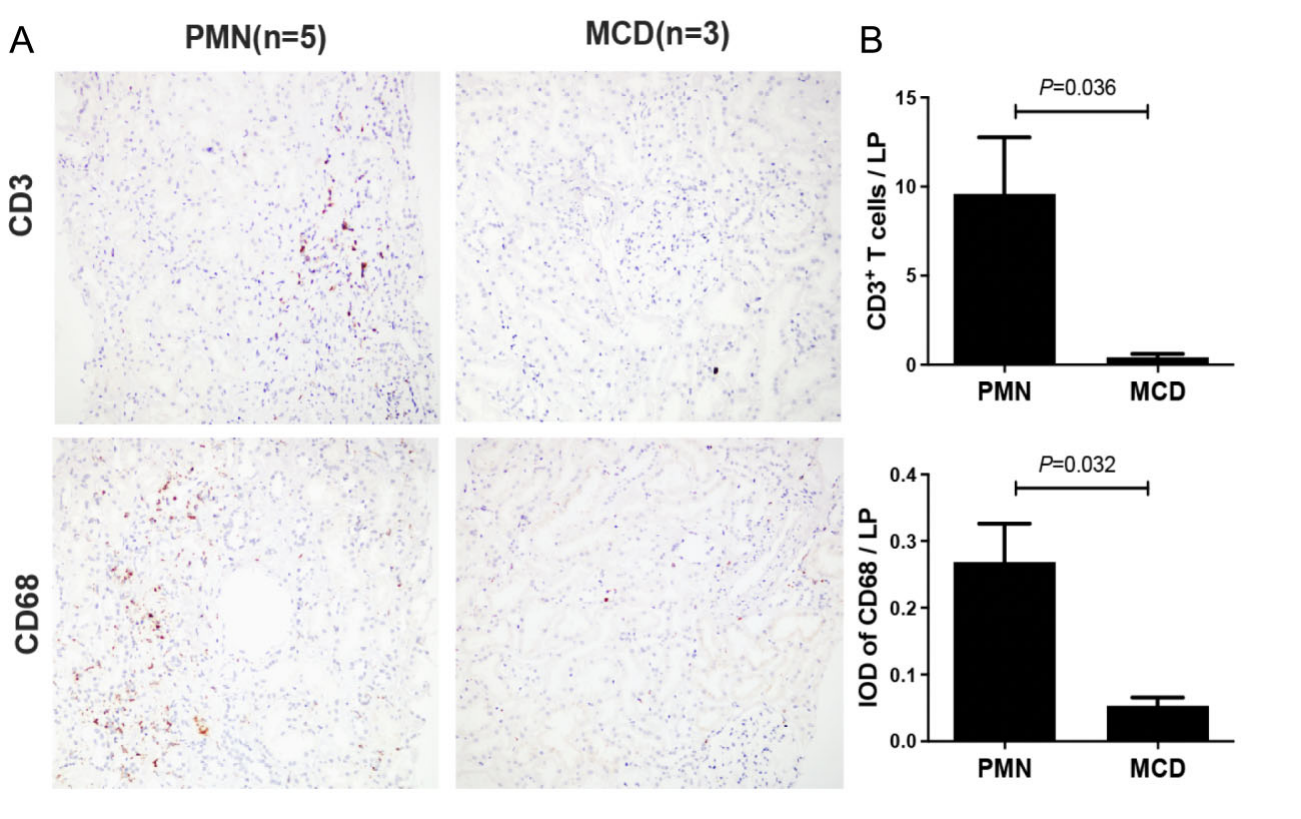
Infiltration of T cells and macrophages in the kidney of patients with PMN validated by immunohistochemistry. (**A**) Microscopy images showing staining of T cells and macrophages in the kidney of patients with PMN (*n* = 5) and MCD (*n* = 3). (**B**) Quantitative analysis of kidney infiltration of T cells and macrophages.

Statistical analysis of cell composition within each group unveiled the following conclusions: (i) Evident elevation in T-cell proportion was found among both renal tissue and urine samples of PMN patients when compared with that of the two healthy controls; and (ii) within urine cells of PMN patients, a notable increase in the proportion of immune cells was evident, particularly characterized by a substantial rise in myeloid cells and neutrophils (Fig. 3C and D). The typical markers denoted 17 cell types are illustrated in Fig. 3E. Immunochemical staining confirmed that kidney infiltrations of T cells and macrophages in patients with PMN were significantly increased (Fig. 4), and mainly distributed in the tubulointerstitial areas (Fig. 4). Taken together, these results suggest that immune cells are abnormally infiltrated into renal tissue in PMN patients and their urine samples manifest similar alteration as well.

We then carried on a comprehensive analysis including DEGs analysis: both up-regulated and down-regulated genes across kidney and urine samples were prioritized (Supplementary data, Fig. S4B). Within kidney tissues, the up-regulated genes are correlated to the human leukocyte antigen (HLA) system and the heat shock protein family (Supplementary data, Fig. S4B). Genes related to the metallothionein family are downregulated in PMN patients compared with healthy controls (Supplementary data, Fig. S4B). In the urine samples, upregulated genes in PMN patients encode various inflammatory factors, including genes associated with the nuclear factor-κB signaling pathway, chemokines and interleukins (Supplementary data, Fig. S4C). Furthermore, HLA family genes were also upregulated in urine samples of PMN patients, accompanied by downregulation of genes within the metallothionein family (Supplementary data, Fig. S4C). GO pathway enrichment analysis of the up-regulated genes suggests that metal ion interactions and major histocompatibility complex (MHC) molecular functions are enriched within both kidney and urine samples (Supplementary data, Fig. S5).

### scRNA-seq analysis of kidney tissue and urine samples revealed profoundly altered cell–cell communications in PMN patients

**Figure 5:**
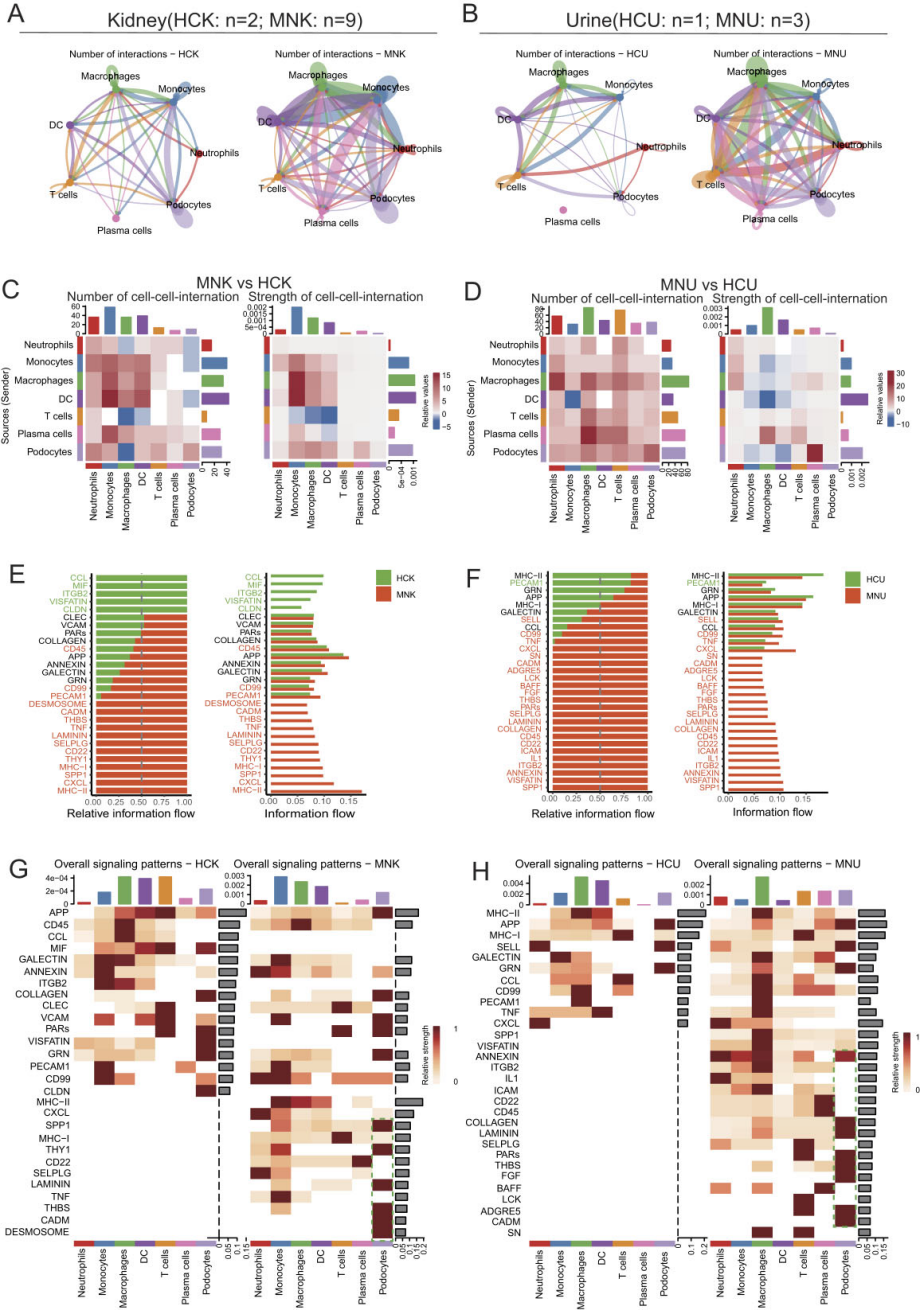
Altered cell–cell interactions and gene expression in podocytes and immune cells from the urine and kidney tissue of patients with PMN. (**A, B**) Circle plots illustrating the overall cell–cell interaction patterns between podocytes and immune cells in kidney (nine patients and two healthy controls) and urine (three patients and one healthy control) scRNA-seq dataset from PMN patients and healthy controls. (**C, D**) Heatmaps showing the communication scores of receptor–ligand interactions in kidney and urine scRNA-seq dataset from two groups. (**E, F**) The bar plots displaying the differences in cell–cell communication information flow between PMN patients and healthy individuals in kidney and urine scRNA-seq dataset. (**G, H**) Heatmap plots showing the differences in the overall signal patterns between these two groups. Notice that the lower parts of MNK and MNU are quite different from HCK and HCU, respectively. Especially when podocytes are considered, signals like *SPP1, THY1, LAMININ, THBS, CADM* and *DESMOSOME* are only indicated in MNK; signals like *ANNEXIN, COLAGEN, LAMININ, PARS, THBS, FGF, ADGRE5* and *CADM* are only indicated in MNU.

Quantification of cell–cell communication may profile important interplays between individual cells within a biological system. To decipher the dynamics of cellular responses, intercellular crosstalk and the altered signaling pathways in patients with PMN, we analyzed the scRNA-seq datasets of kidney and urine using CellChat and NicheNet for cell communication analysis [23, 24]. We observed a remarkable surge in cell–cell interactions within renal tissues and urine samples of patients, setting them apart from that in healthy controls (Figs 5A and 5B). The increased complexity (number of interactions) and intensity (strength of interactions) of intercellular networks may underscore the transformative nature of the PMN disease. For example, in renal tissues, macrophages and dendritic cells exhibited a robust enhancement in signaling towards monocytes among PMN patients compared with healthy controls (Fig. 5C). In contrast, signaling from T cells to monocytes, macrophages and dendritic cells is decreased in PMN patients compared with healthy controls (Fig. 5C). In urine samples, a distinctive elevation in signaling emanating from podocytes to plasma cells was detected in the scRNA-seq datasets of PMN patients compared with healthy controls (Fig. 5D). Conversely, signals from dendritic cells that typically received messages from macrophages displayed a discernible weakening in the PMN patients (Fig. 5D). Intriguingly, we revealed a range of novel signaling molecules within the kidney of patients, including *TNF, CXCL, MHC-I, MHC-II* and others (Fig. 5E). When considering podocytes, the major injured cell type of PMN pathophysiology [30, 31], we detected notable upregulation of signaling molecules including *SPP1, THY1, LAMININ, THBS, CADM* and *DESMOSOME* (Fig. 5E and G; green arrows in 5G). Interestingly, the altered cell–cell landscape in urine mirrored these active states of signaling molecules in PMN patients, with podocytes displaying distinct presence of molecules such as *COLLAGEN, LAMININ, PARs, THBS, FGF, LCK, ADGRE5* and *CADM* (Fig. 5F and H; green arrows in 5H). Taken together, the cell–cell communication analysis based on the scRNA-seq datasets revealed a surged alteration in inflammatory myeloid cells and podocytes.

### Consensus biomarker extraction across blood, kidney and urine in PMN

**Figure 6:**
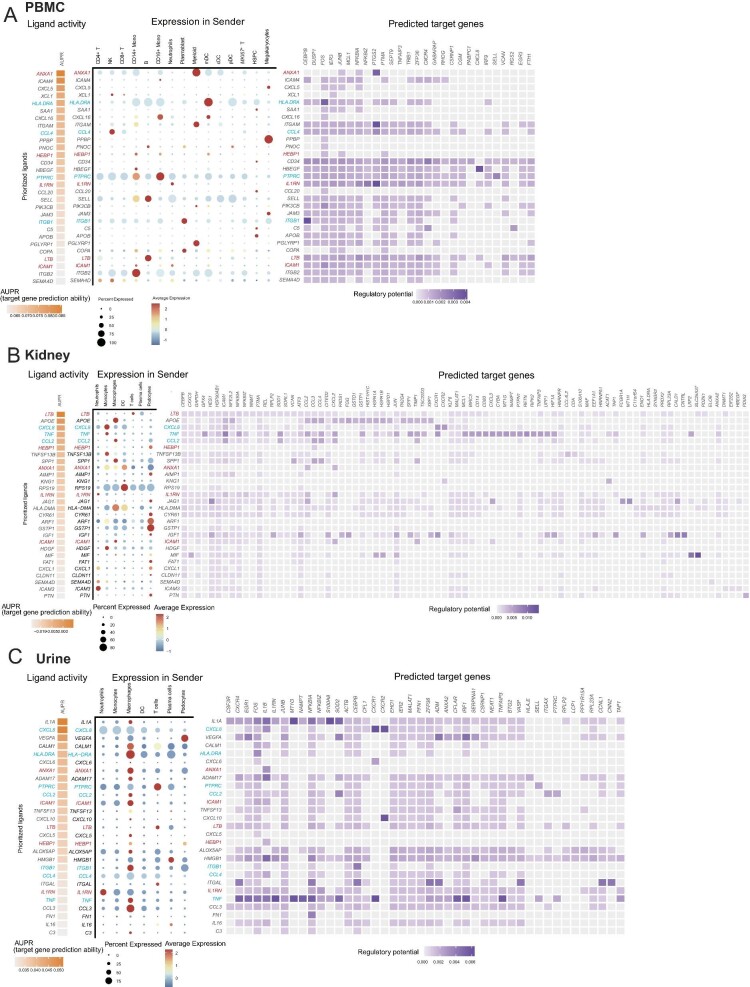
Consensus of altered cell–cell interactions and gene expression in the PBMC, kidney tissue and urine samples of PMN patients. (**A**–**C**) Cell communication profiles analyzed by the nichenetr algorithm, illustrating cell interactions in the PBMC, kidney and urine. In each plot, the bar chart (left panel) is ordered by ligand activity; the spot diagram (middle panel) shows ligand expression of sender cells; the heatmaps (left panel) depict the predicted expression of target genes. The consensuses of the altered pathways are highlighted in red including: *ANXA1, HEBP1, LTB, IL1RN* and *ICAM*.

**Figure 7:**
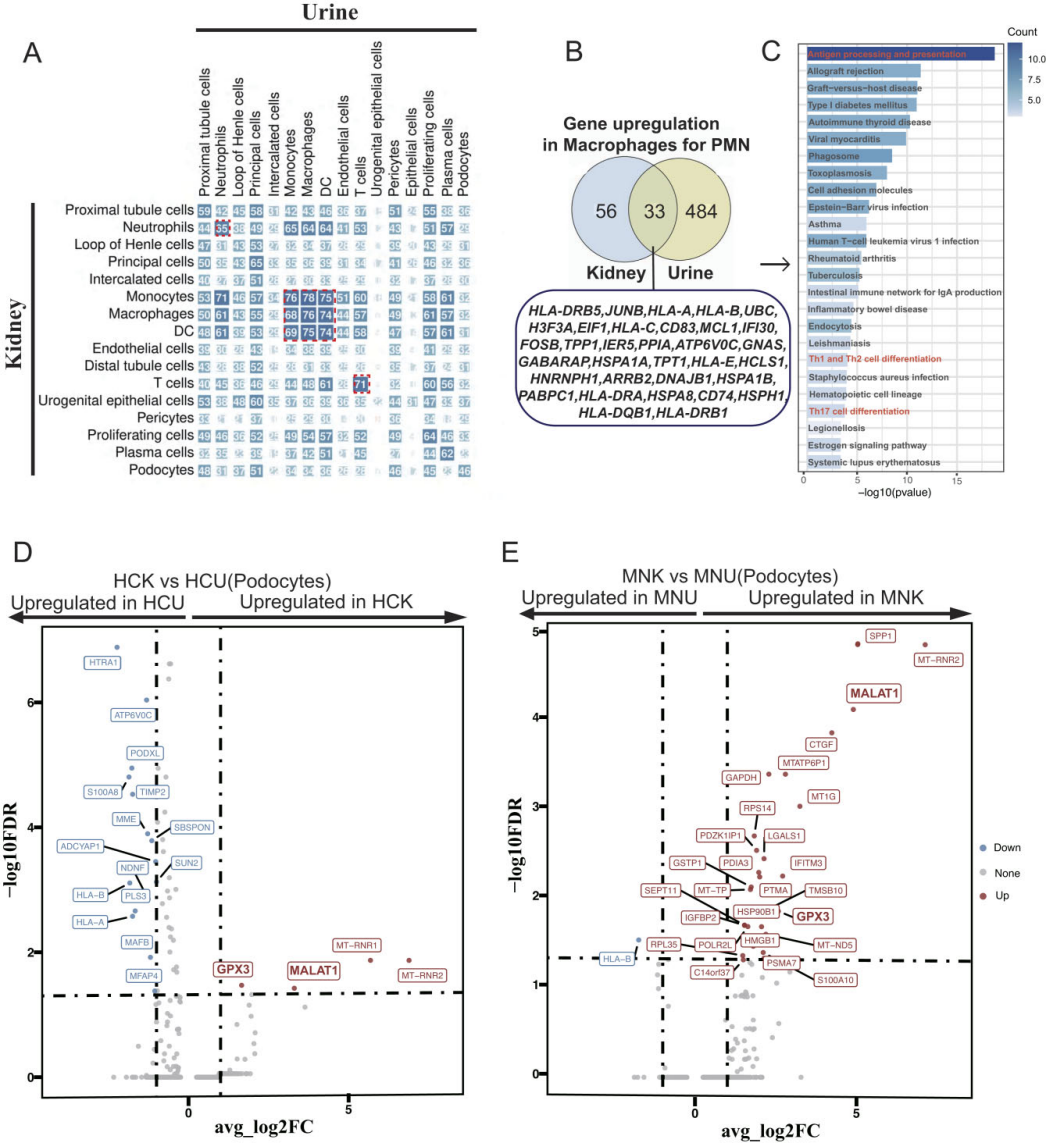
Correlations between kidney and urine samples in scRNA-seq datasets and comparison of the altered gene expression in podocytes from kidney and urine. (**A**) Correlation plot for gene expression of each 500 most variable expressed genes in urine (columns) and kidney cell types (rows). Gene size (in numbers) and color (from dark red to dark blue) represent the Spearman ρ (×100) with all *P* < .001. As indicated by the dashed lines, the correlation of immune cells between the two tissues is relatively highest in both color and gene size in the entire correlation analysis panel. (**B**) Venn diagram showing the upregulated genes in the macrophages from patients’ kidney and urine. In total 33 genes are identified co-upregulated in MNK and MNU. (**C**) KEGG enrichment analysis suggests pathways enriched by the 33 co-upregulated genes. The 33 genes are associated with several immune response (highlight in red). (**D, E**) When podocytes were considered, we did the volcano plots showing upregulated and downregulated genes compared in the comparison as indicated (HCU vs HCK; or MNU vs MNK).

Three different types of samples of PMN patients were collected in the study—blood, kidney biopsy and urine samples—facilitating in identifying of consensus biomarkers for this autoimmune disease. We aligned cell–cell communication analysis across these three distinct tissues by NicheNet analysis. We revealed the five consensus signaling molecules: Leukotriene B (*LTB*), Heme Binding Protein 1 (*HERP1*), Annexin A1 (*ANXA1*), Interleukin 1 Receptor Antagonist (*IL1RN*) and Intercellular Adhesion Molecule 1 (*ICAM1*) (Fig. 6A–C). As urine samples are tightly correlated to kidney pathologies, we analyzed associations of gene expression of various cell types between kidney and urine tissues in PMN. Indeed profiling the gene expression landscapes unveiled a striking molecular resemblance (consensus) between renal and urinary immune cells by the BioNERO tools [32]. Remarkable coherence in T cells and myeloid cells gene expression was also evident through pairwise correlation analysis and gene expression comparisons (Fig. 7A). We further revealed 33 co-upregulated genes in macrophages from urine and kidney of patients with PMN compared with that in healthy controls: (i) MHC class genes (including *HLA-DRB5, HLA-A, HLA-B, HLA-C, HLA-E, HLA-DRA, HLA-DQB1, HLA-DRB1*); (ii) transcription factors (*JUNB, EIF1, CD83, FOSB, HCLS1, HNRNPH1, ARR2B*); and (iii) genes related to immune response and antigen presentation (*IFI30, ATP6V0C, TPT1, CD74*) (Fig. 7B and C). These results indicated that biomarkers from urine partially reflect alterations in the kidney of PMN.

To further analyze changes in podocytes, we carried on a comparative differential gene analysis, juxtaposing healthy individuals’ urine and kidney samples. We subjected patients’ samples to the same procedure to dissect podocyte transformations before and after shedding (Fig. 7D and E). In both healthy controls and patients with PMN, *GPX3* and *MALAT1* in the podocytes from kidney were highly expressed compared with those from urine (Fig. 7D and E), indicating their potential roles in protecting the podocytes from shedding to the urine.

### Immune repertoire analysis showed different clonal landscapes within patients with PMN

**Figure 8:**
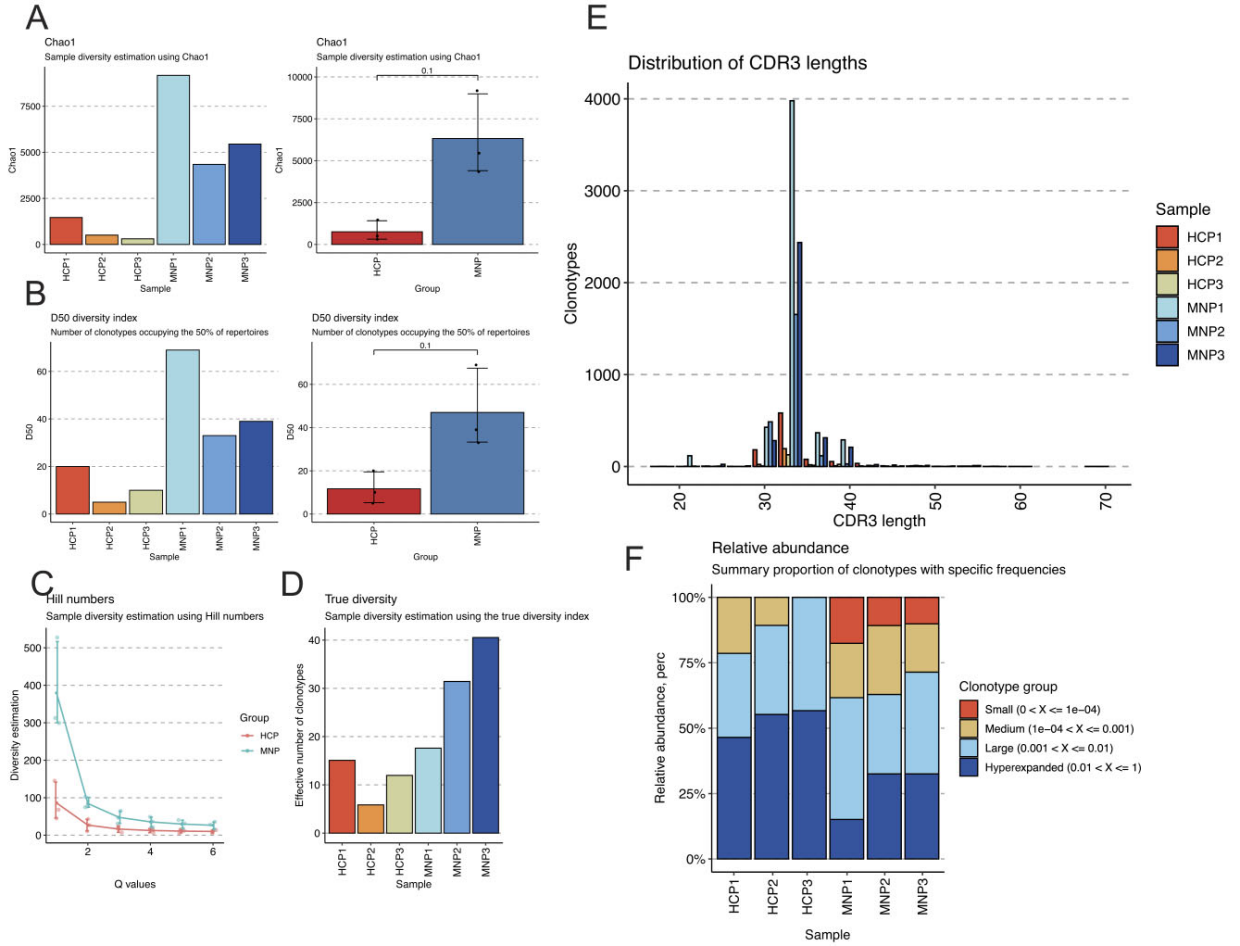
PMN patients exhibited an altered landscape in the immunological repertoire. (**A**–**D**) The clonal diversity of CDR3 sequences is evaluated by the Chao1 index, D50 diversity index, Hill numbers and true diversity for each individual sample and qualified between each group (three patients and three healthy controls). (**E**) Bar graphs showing the distribution of CDR3 length in each sample. (**F**) Bar plots showing the summary proportion of major clonotypes with specific frequencies.

Autoimmune diseases may have distinct TCR/BCR clonal landscape [33, 34]. Our recent study on IgG4-related diseases (IgG4-RD) revealed that IgG4-RD had increased CDR3 diversity and expansion of certain clone types [35]. We performed similar immune repertoire analysis in the PBMC samples of PMN patients and healthy controls (Fig. 8, Supplementary data, Figs S6 and S7). Elevated clonal diversity was readily observed within PMN patients by quantifying diverse indices (Fig. 8A–D). The diversified clonotypes with CDR3 are ranging from 30 to 40 amino acids (Fig. 8E). Overall, CDR3 clone types in small length has increased portion in PMN patients (Fig. 8F), indicating active *de novo* TCR/BCR production in PMN disease. Unlike IgG4-RD, each PMN patient exhibited a distinct immune landscape thus no shared clonotypes were identified in the study (Supplementary data, Fig. S7E). Taken together, the immune repertoire study on PBMC scRNA-seq datasets revealed diversified clone types of TCR/BCR, but no shared CDR3 sequences were identified in the mini-cohort study.

## DISCUSSION

PMN is an autoimmune disease with specific and profound damage to kidney. Comprehensive studies with new technologies are demanded to dissect the pathophysiology alterations and identify new therapy targets at cell and molecular levels. To our knowledge, the present study is the first to systematically profile the landscapes of PMN at the single cell transcriptome resolution using blood, kidney and urine samples. We profiled landscape alterations in cell composition of three tissues, along with cell–cell communication, signaling pathways and repertoire in the immune cells and podocytes.

By performing scRNA-seq of the PBMC from patients with PMN, we found that the proportion of B cells and plasma cells was significantly higher than that of healthy controls, which was consistent with previous studies [28, 29, 36], in accordance to the knowledge that B cell plays a key role in the development of MN [37], and depleting the circulating B cells by CD20 antibody has become one of the efficacious treatment [4, 29, 38].

Compared with the healthy control, cell communication analysis found the *APRIL* signaling showed an obvious enhancement, especially when plasma cells act as the receiving cells. In addition, the enhanced output *APRIL* signaling might be from monocytes. While the elevation of *BAFF* signaling was not pronounced, both *APRIL* and *BAFF* are key players in B and plasma cell survival and differentiation [39, 40]. Previous studies showed the circulating levels of *BAFF* and *APRIL* were significantly higher than that of healthy controls, and correlated to clinical remission after treatment [41, 42]. These results indicated that *APRIL/BAFF* signaling might be the important regulator involved in the pathogenesis of PMN, underlying a potential treatment target. A further functional investigation will warrant that. Notably, few neutrophils, which might be low density neutrophils associated with autoimmune disorder [43–46], could be detected in the PBMC from both patients and healthy controls. Though the neutrophils proportion was significantly increased, further investigation will be needed to evaluate the significance of low-density neutrophils in PMN. In addition, the results from the current study of an increased proportion of *MKI67*^+^ T cells and myeloid cells, as well as a significant enrichment of inflammatory signaling pathway provided an explanation that autoimmune response, especially the activation of antigen-specific T and B cells, results in the initiation of PMN.

To further depict the characteristics of the autoimmune reactive T/B cells in the circulation of patients with PMN, we also analyzed the immune repertoire based on the data from PBMC. We found patients with PMN had an elevated clonal diversity. Although there were no shared top clonotypes found among the patients in our study, Su *et al.* found increased *IGHM, IGHD* and *IGHE* frequencies and decreased *IGHA* and *IGHG4* frequencies in the PBMCs of patients with PMN, and *IGHV3-66* showed reactive to the immune-suppressive treatment [47]. With regard to the TCR repertoire, Zhang *et al.* found the VJ combination diversity of patients was significantly lower than that of healthy controls, and the usage patterns of *V*β and *J*β genes were skewed in patients with PMN [48]. The previous studies and the results in our study indicated that there is an altered immune response associated with PMN. Recently, the T-cell epitopes of PLA2R in patients with PMN have been mapped in detail [11]. Dominant conformational epitopes of PLA2R in PMN have also been identified [49–51]. Further investigation based on a large PMN cohort is required to identify disease-specific TCR/BCR clonotypes reactive to the T/B cell epitopes on the PLA2R antigen.

The main clinical manifestation of patients with PMN arises from the direct injury on kidney. Podocyte is recognized as the target cell of anti-PLA2R autoantibody. In addition, T cells and macrophages are the main infiltrating cells in PMN [52, 53]. For example, in some patients, even tertiary lymphoid structure could be observed which were associated with the severity of clinical features [54]. T cells and myeloid cells were the dominant immune cells, which was also found in the report of Xu *et al.* by performing kidney scRNA-seq of patients with PMN [16]. These clinical observations were consistent with our scRNA-seq analysis.

Cell communication analysis revealed that interactions between cells in the kidney of PMN patients were enhanced, especially cells among podocytes, macrophages, DCs and monocytes. We also identified a series of molecules that might be involved in the signaling communication associated with kidney injury. In particular, some of them were identified in the podocyte, which is the key pathology-driven cell of PMN. By comparing the glomerular gene expression between MN and other glomerulonephritis, hundreds of genes were found significantly differentially expressed in MN, and 43% of these genes were most highly expressed in the podocytes [31]. These findings accordingly identify podocytes as the key player in the development of MN and might facilitate the exploration of new therapy targeted podocytes.

Urine samples have long been speculated as the mirror of kidney. A previous study found almost all the kidney cell types including podocytes, tubular cells, macrophages and lymphocytes could be detected in urine by scRNA-seq [55, 56]. In the current study, we found myeloid cells and T cells were the dominant cell types except abundant cells from urinary tract, comparable to the study reported by Liu *et al*., who found urinary macrophages accounted for the main myeloid cells and associated with clinical remission [17]. Obvious correlations in myeloid and T cells from kidney and urine were also found, indicating these cells in urine were from kidney. In the patients with lupus nephritis, urinary samples also had a higher frequency of myeloid cells, and gene expression in corresponding urine and kidney clusters were highly correlated [57]. Podocytes could also be identified in urine by scRNA-seq in others’ and our study, suggesting that podocytes could be shed into the urine [58, 59]. Common signal molecules related to podocytes were also found in kidney and urine of our results. By scRNA sequencing of the urine cells of patients with focal segmental glomerulosclerosis (FSGS), the signature genes of urinary immune cells and podocytes were also found highly expressed in FSGS biopsies [60], which also reflected the diagnostic and prognostic role of urinary cell in glomerulonephritis. Nonetheless, these results provide noninvasive insight into PMN and will contribute to the better monitoring of disease development.

By comprehensively analyzing the cell–cell communication across the PBMCs, kidney and urine samples, we found five conserved signaling molecules including *LTB, HERP1, ANXA1, IL1RN* and *ICAM1*. These signaling molecules might be involved in the signal transduction in the circulation and kidney that can induce the development of PMN. Further experimental validations are demanded to warrant them as reliable biomarkers or treatment targets for patients with PMN.

There are still some limitations in the current study. Due to the difficulty to collect the samples from patients, the sample size was small and further experiments to verify our findings derived from scRNA-seq have not been performed.

In conclusion, by profiling the transcriptomic landscapes of blood, kidney and urine of patients with PMN at the single cell resolution, we characterize B cells and plasma cells in the circulation were the prominent cell type and *APRIL* might be the potential regulator for the autoimmune disease. T cells and myeloid cells were the main infiltrating cells in the kidney and might contribute to the podocyte injury. Urinary scRNA-seq provided a noninvasive insight into the diagnosis and monitoring of PMN as it is reminiscent of the cell landscape with kidney. Our present study shows a comprehensive analysis across PBMCs, kidney and urine, and facilitates the exploration of the pathophysiology and therapeutic targets of PMN.

## Supplementary Material

sfae168_Supplemental_Files

## Data Availability

The raw sequencing data from this study have been deposited in the Genome Sequence Archive in BIG Data Center (https://bigd.big.ac.cn/), Beijing Institute of Genomics (BIG), Chinese Academy of Sciences, under the accession number: PRJCA021361 (the datasets will be available to the public once the manuscript in press).
