# Supplementary material for: Integrative profiling of untreated primary membranous nephropathy at the single-cell transcriptome level
Source: Clin Kidney J. 2024 Jun 14;17(7):sfae168. doi: 10.1093/ckj/sfae168 (PMC11255483; doi:10.1093/ckj/sfae168)

Supplemental Figure 3

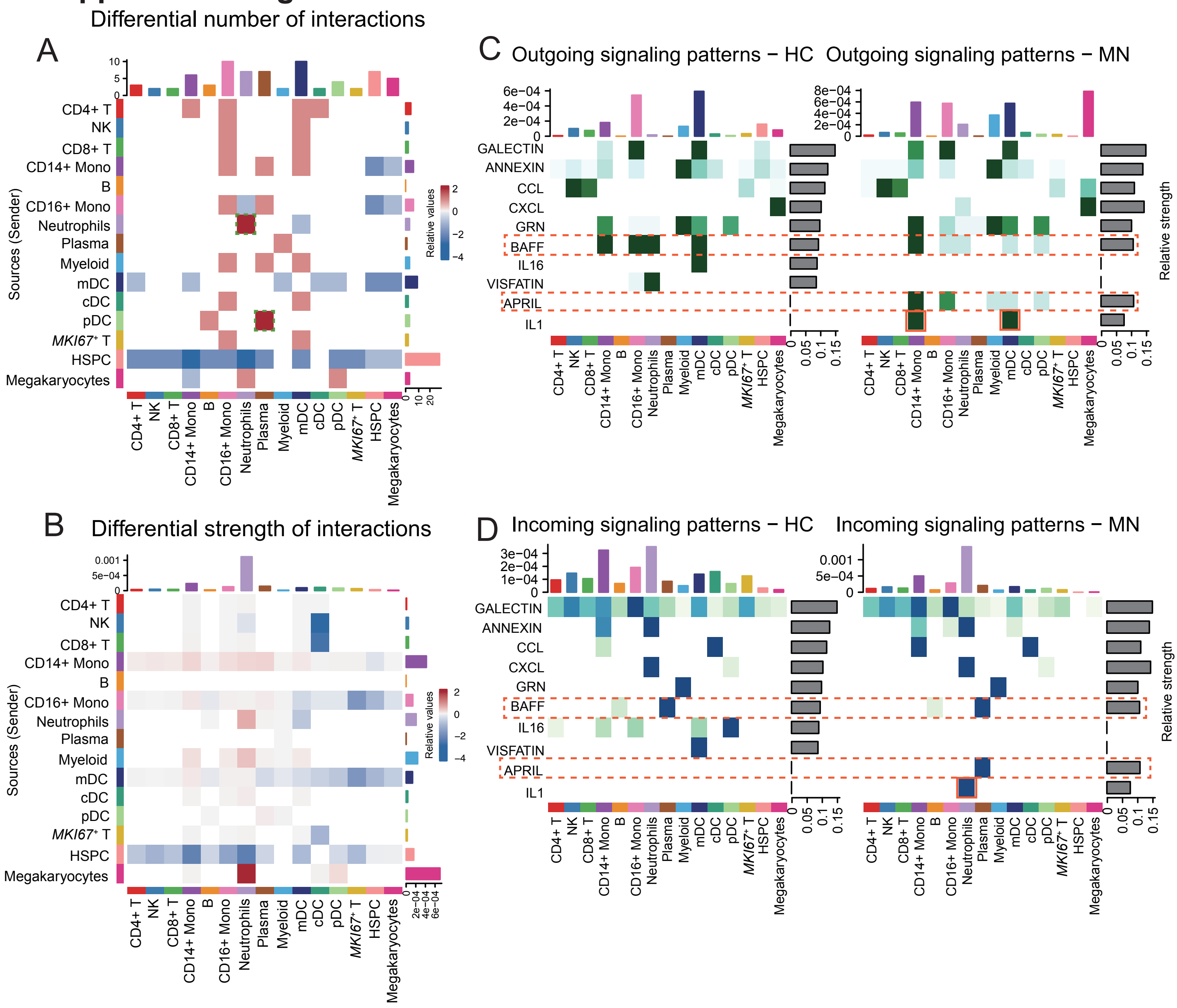

Supplement: sfae168_Supplemental_Files [file sfae168_supplemental_files.zip › Supplemental Figure 3.pdf]
