# Supplementary figures and images for: Integrative profiling of untreated primary membranous nephropathy at the single-cell transcriptome level
Source: Clin Kidney J. 2024 Jun 14;17(7):sfae168. doi: 10.1093/ckj/sfae168 (PMC11255483; doi:10.1093/ckj/sfae168)

**Supplemental Figure 1**

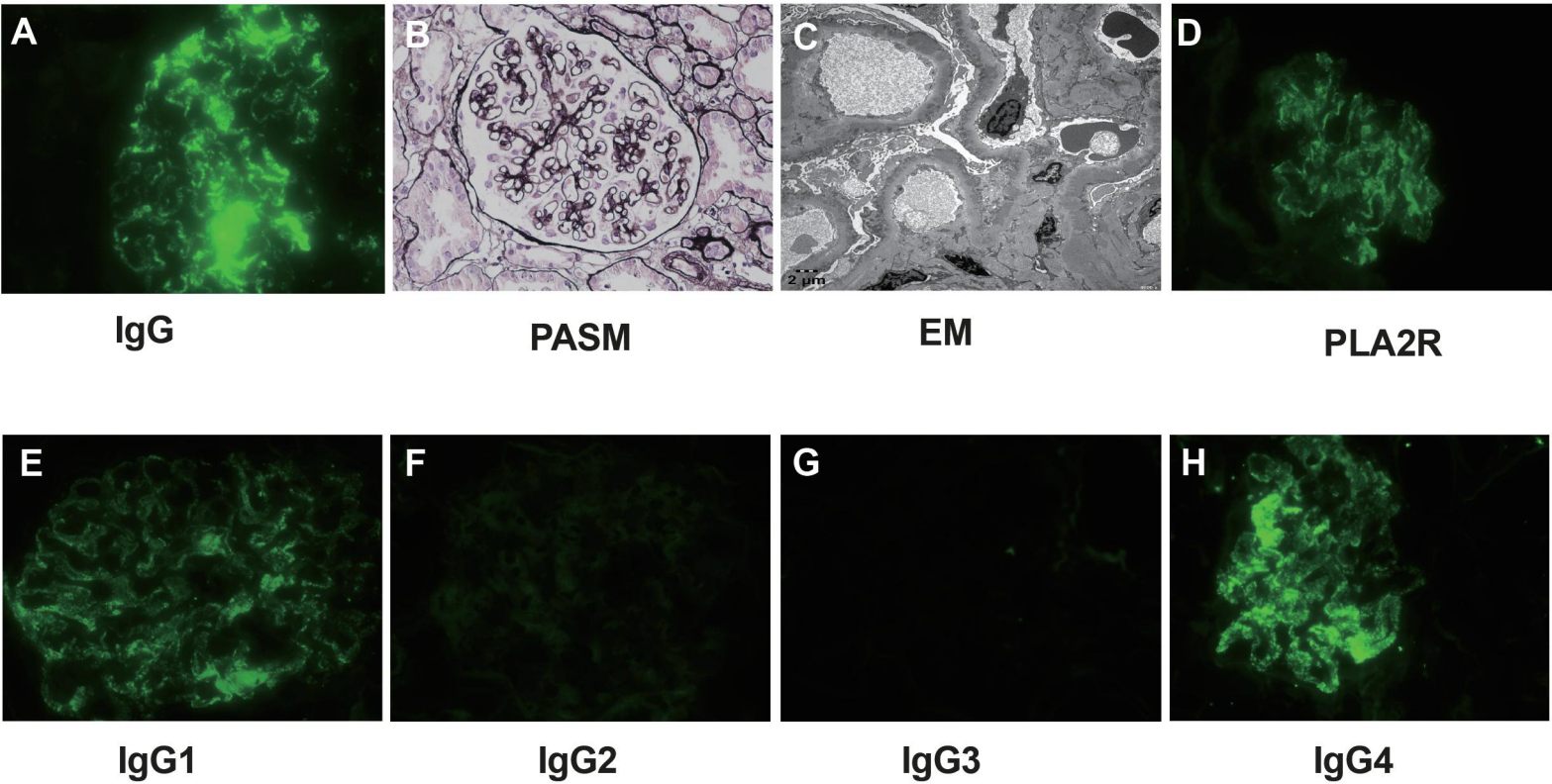

Supplement: sfae168_Supplemental_Files [file sfae168_supplemental_files.zip › Supplemental Figure 1.pdf]

# Supplemental Figure 2

A

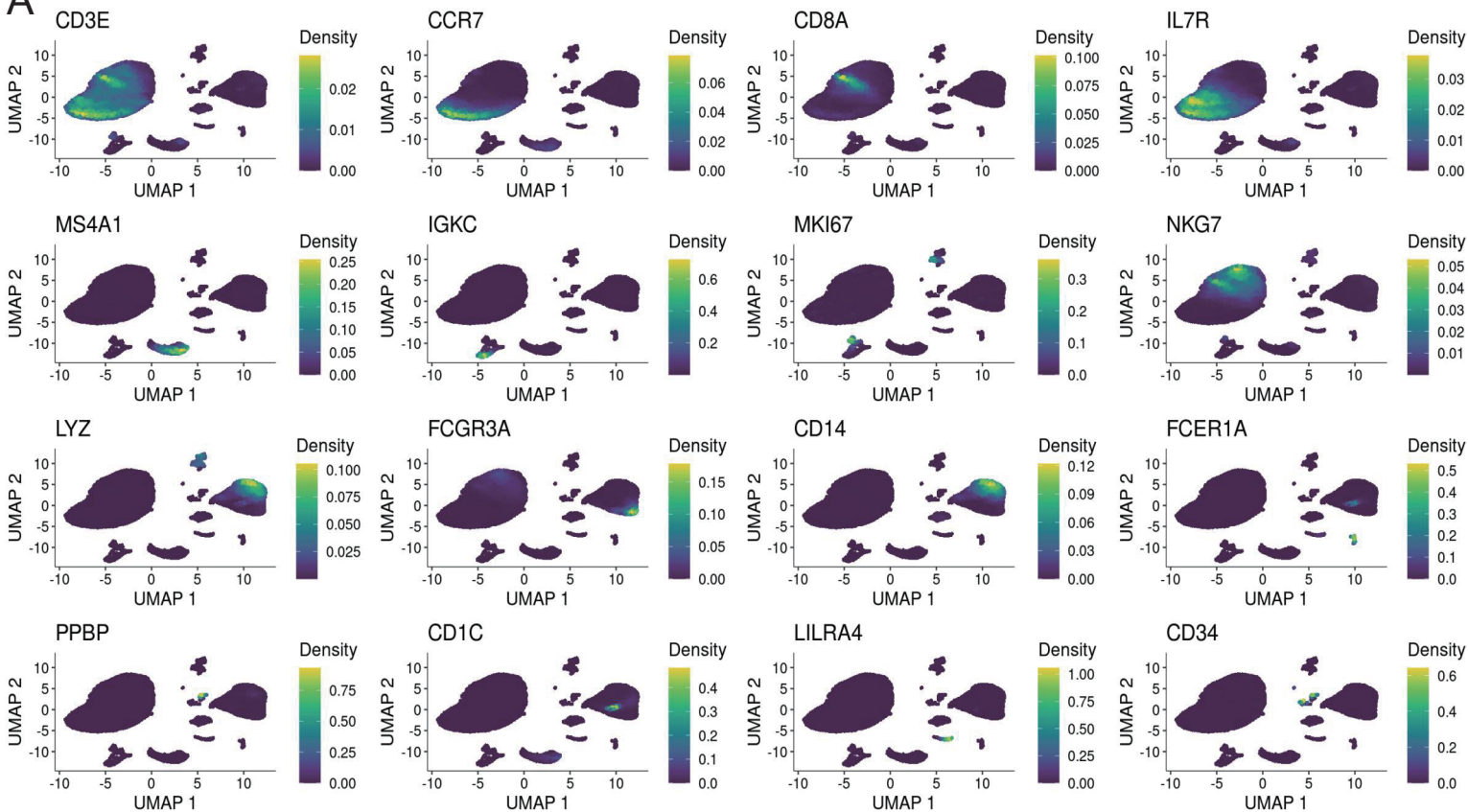

B

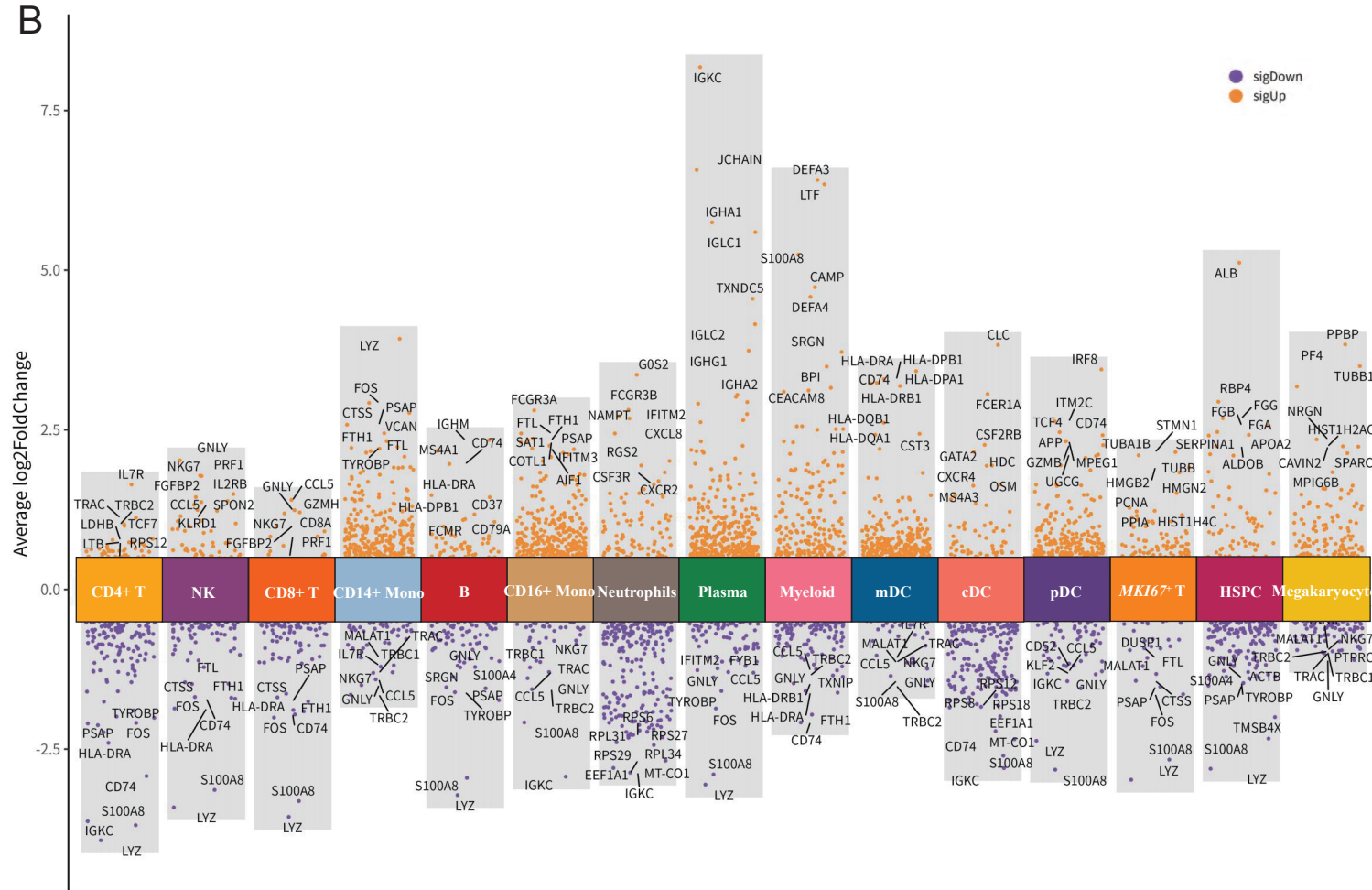

Supplement: sfae168_Supplemental_Files [file sfae168_supplemental_files.zip › Supplemental Figure 2 .pdf]

## A

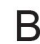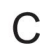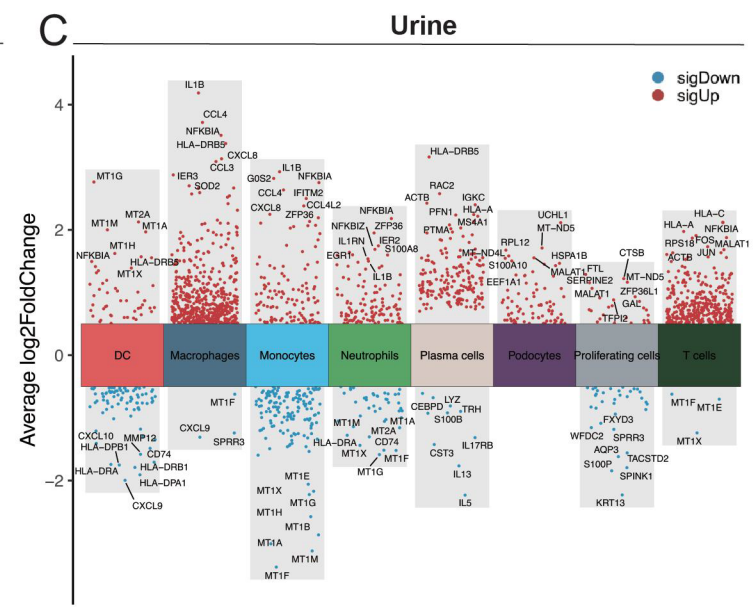

Supplement: sfae168_Supplemental_Files [file sfae168_supplemental_files.zip › Supplemental Figure 4.pdf]

# Supplemental Figure 5

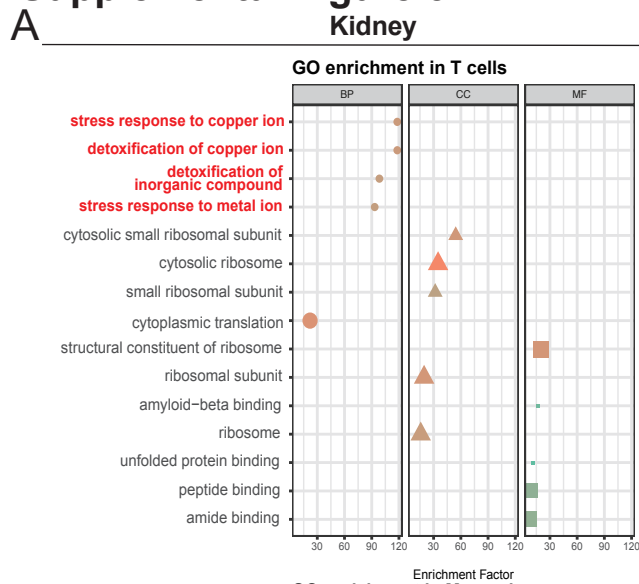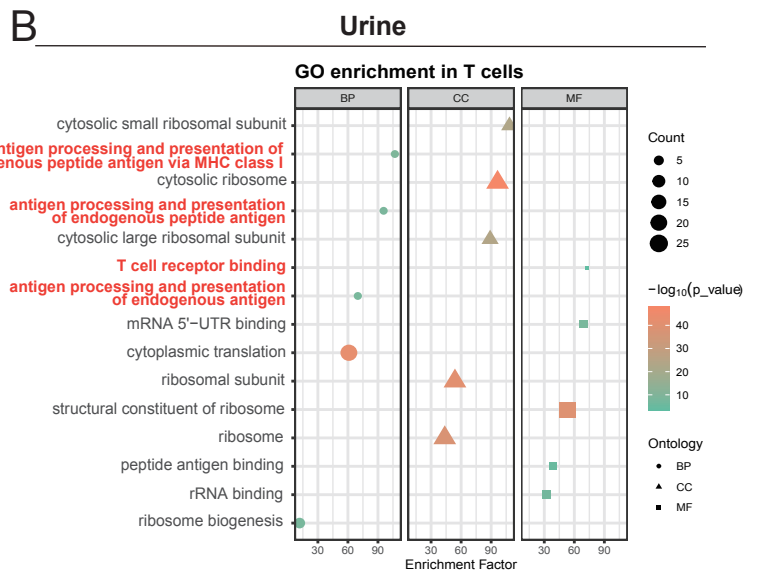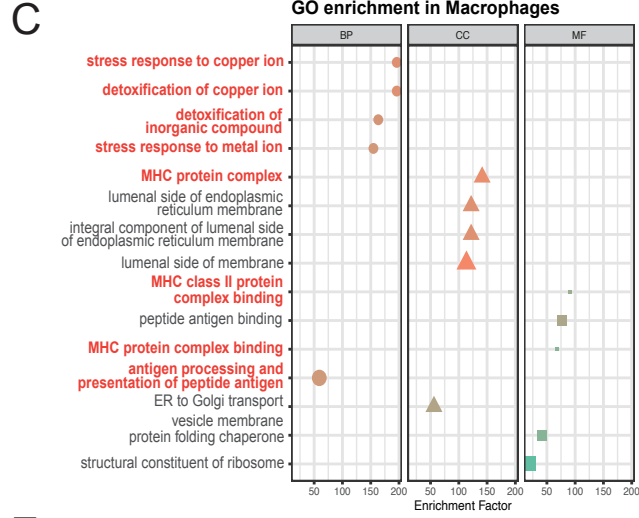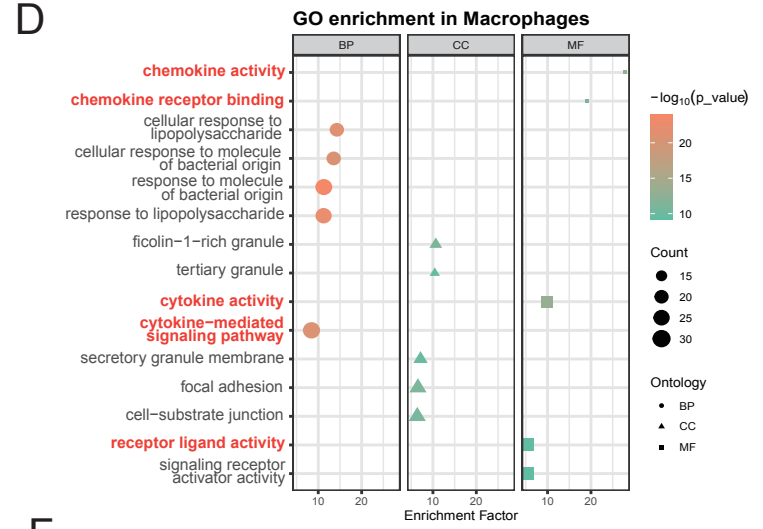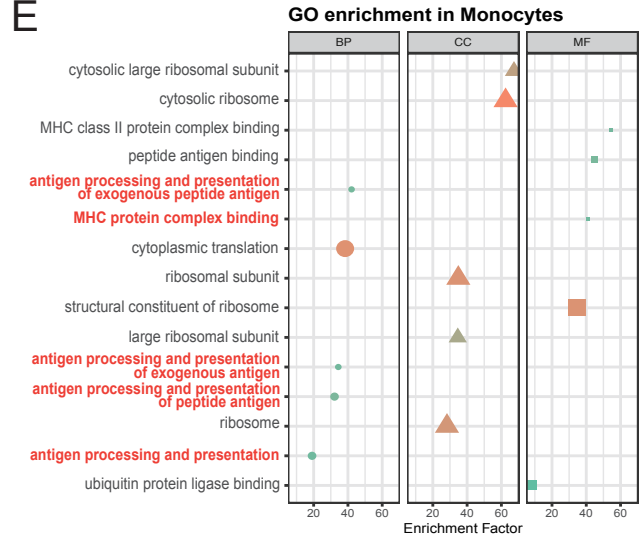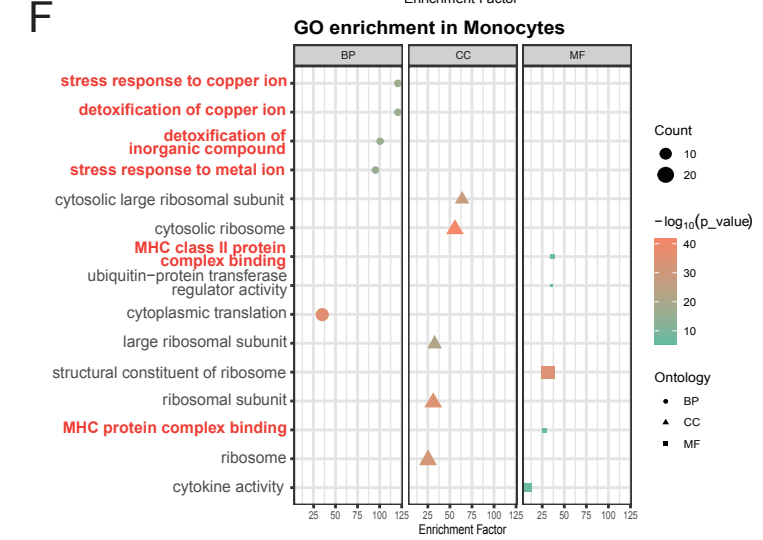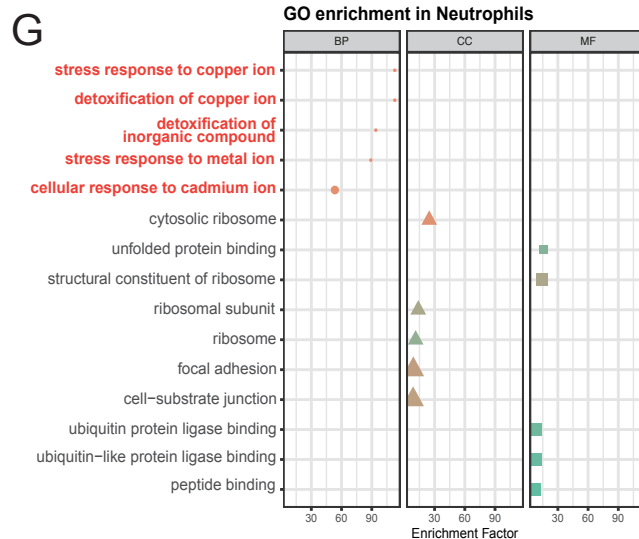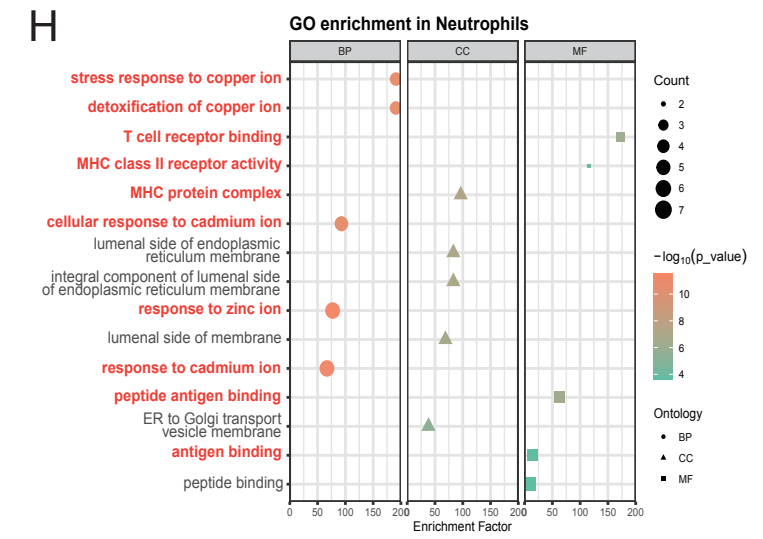

Supplement: sfae168_Supplemental_Files [file sfae168_supplemental_files.zip › Supplemental Figure 5.pdf]

## A Gene usage in BCR

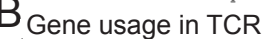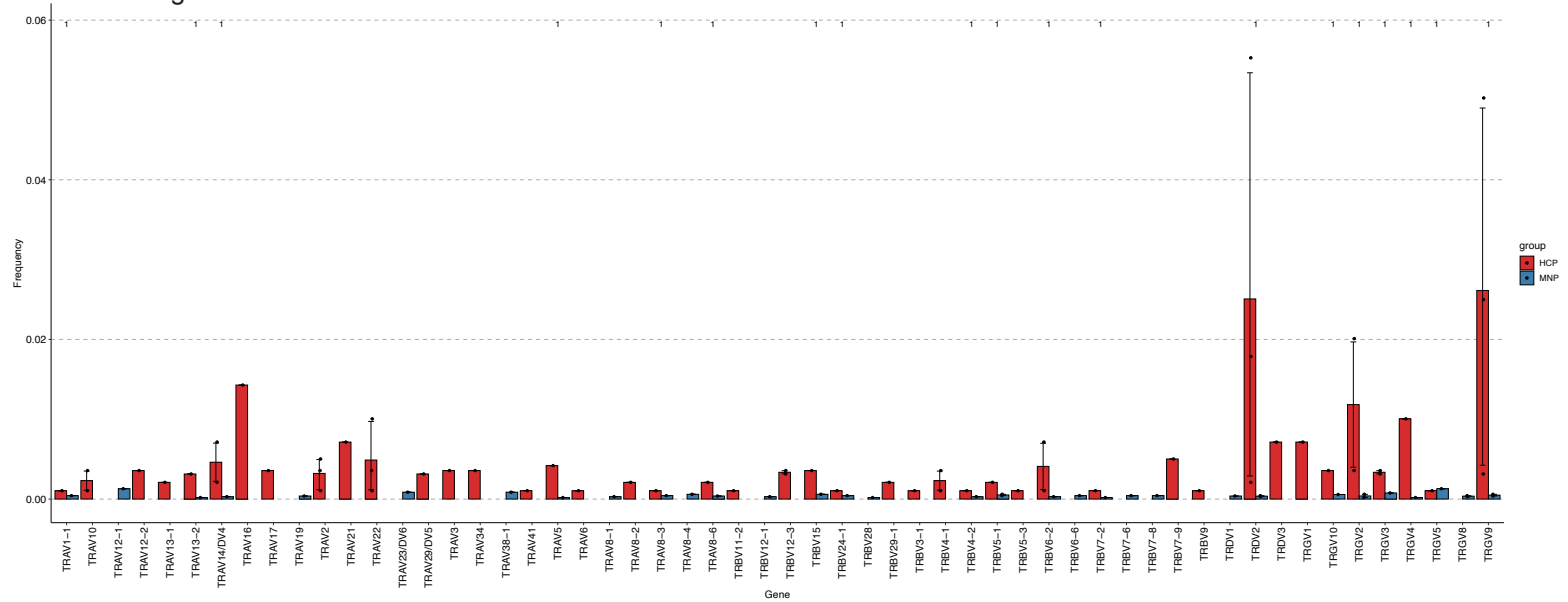

Supplement: sfae168_Supplemental_Files [file sfae168_supplemental_files.zip › Supplemental Figure 6.pdf]

Supplemental Figure 7

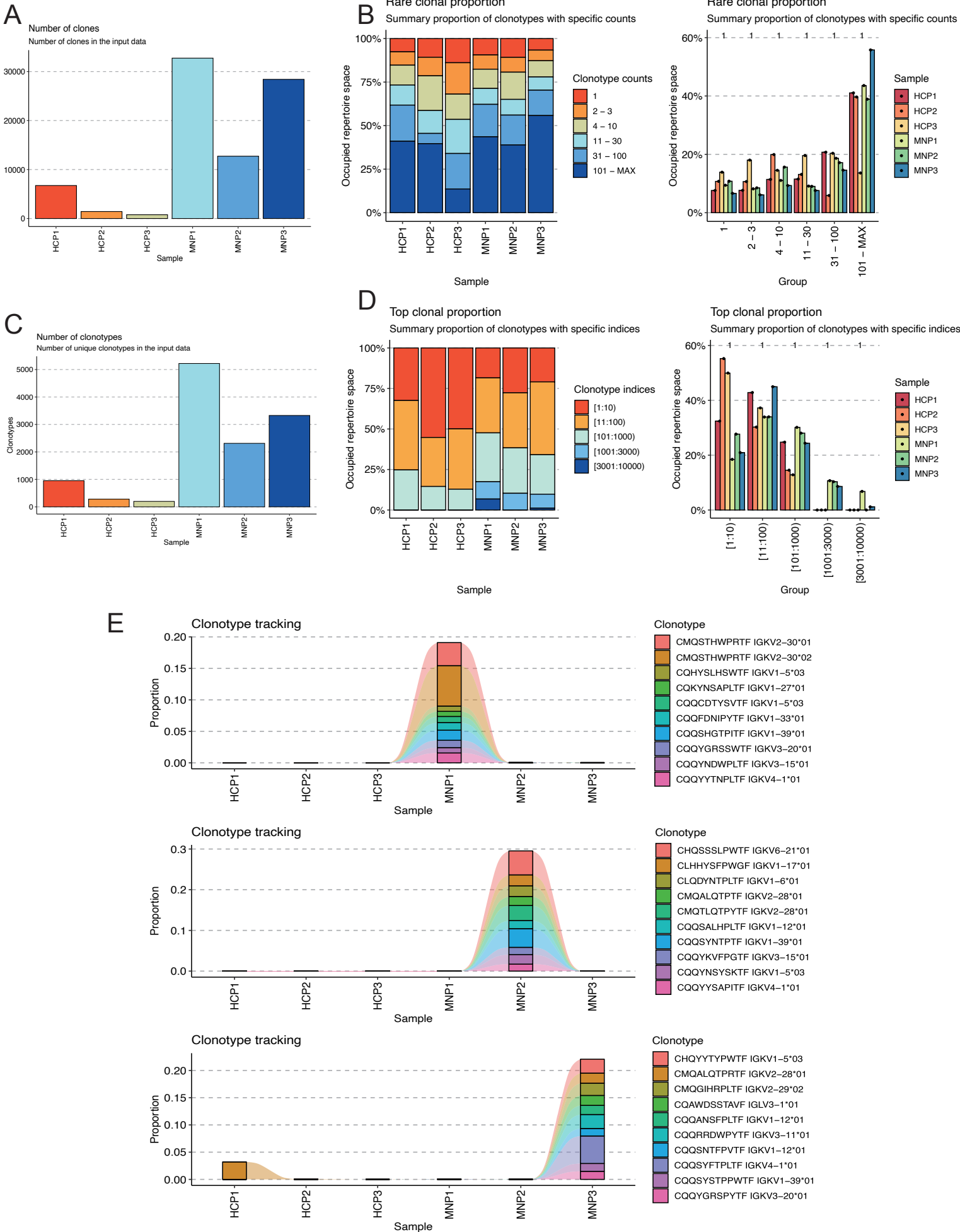

Supplement: sfae168_Supplemental_Files [file sfae168_supplemental_files.zip › Supplemental Figure 7.pdf]
